# Therapeutic CFTR Correction Normalizes Systemic and Lung-Specific S1P Level Alterations Associated with Heart Failure

**DOI:** 10.3390/ijms23020866

**Published:** 2022-01-14

**Authors:** Franziska E. Uhl, Lotte Vanherle, Frank Matthes, Anja Meissner

**Affiliations:** 1 Department of Experimental Medical Sciences, Lund University, 221 84 Lund, Sweden; franziuhl82@gmail.com (F.E.U.); lotte.vanherle.8386@med.lu.se (L.V.); frank.matthes@med.lu.se (F.M.); 2 Wallenberg Centre for Molecular Medicine, Lund University, 221 84 Lund, Sweden

**Keywords:** heart failure, sphingosine-1-phosphate, cystic fibrosis transmembrane regulator, inflammation, lung

## Abstract

Heart failure (HF) is among the main causes of death worldwide. Alterations of sphingosine-1-phosphate (S1P) signaling have been linked to HF as well as to target organ damage that is often associated with HF. S1P’s availability is controlled by the cystic fibrosis transmembrane regulator (CFTR), which acts as a critical bottleneck for intracellular S1P degradation. HF induces CFTR downregulation in cells, tissues and organs, including the lung. Whether CFTR alterations during HF also affect systemic and tissue-specific S1P concentrations has not been investigated. Here, we set out to study the relationship between S1P and CFTR expression in the HF lung. Mice with HF, induced by myocardial infarction, were treated with the CFTR corrector compound C18 starting ten weeks post-myocardial infarction for two consecutive weeks. CFTR expression, S1P concentrations, and immune cell frequencies were determined in vehicle- and C18-treated HF mice and sham controls using Western blotting, flow cytometry, mass spectrometry, and qPCR. HF led to decreased pulmonary CFTR expression, which was accompanied by elevated S1P concentrations and a pro-inflammatory state in the lungs. Systemically, HF associated with higher S1P plasma levels compared to sham-operated controls and presented with higher S1P receptor 1-positive immune cells in the spleen. CFTR correction with C18 attenuated the HF-associated alterations in pulmonary CFTR expression and, hence, led to lower pulmonary S1P levels, which was accompanied by reduced lung inflammation. Collectively, these data suggest an important role for the CFTR-S1P axis in HF-mediated systemic and pulmonary inflammation.

## 1. Introduction

The bioactive sphingophospholipid sphingosine-1-phosphate (S1P) has been shown to control various cellular events relevant to cardiovascular disease (CVD), including immune cell chemotaxis [1], polarization and cytokine production [2,3,4,5], vascular responsiveness [6,7,8,9,10,11] and barrier function [12,13,14]. Thus, several experimental studies confirmed apparent alterations in S1P metabolism during hypertension [9,15,16], atherosclerosis [17,18], heart failure (HF) [19], and stroke [10,13]. In human disease, plasma S1P levels were associated with increments in systolic blood pressure and several biomarkers of CVD and inflammation [20], disease severity in stroke [21], atherosclerotic plaque inflammation [22], and the failing heart [23].

S1P bioavailability critically depends on the activity of S1P-generating enzymes (sphingosine kinases; SphKs), S1P transporters and S1P degrading enzymes that, together with five receptors (S1PRs), coordinate cell-specific responses. S1P’s vascular and immune system effects have important pathophysiological relevance in CVD, including HF [6,24]. The cystic fibrosis transmembrane regulator (CFTR) has, in particular, emerged as a key element for S1P degradation by mediating S1P import into vascular smooth muscle cells where it controls S1P effects on vascular responsiveness [25,26]. In its role as a component of the S1P degrading pathway, CFTR expression at the plasma membrane is mandatory for functionality. In experimental HF, elevated tumor necrosis factor alpha (TNF-α) levels are responsible for considerable reduction of membrane CFTR expression and, thus, impaired S1P import. As a consequence, S1P degradation is limited and more S1P is available for S1PR-specific signaling, which may be involved in disease progression. Although therapeutic CFTR correction has been shown to mitigate acquired CFTR deregulation and HF-associated target organ damage [11], direct effects on systemic and tissue-specific S1P levels remain unknown.

Specifically in the lung, acquired CFTR dysfunction links to augmented tissue inflammation in the HF lung [27], increased artery vasoconstriction during pulmonary hypertension [28], and correlates with disease severity during chronic obstructive pulmonary disease (COPD) [29,30]. Further, the latter is associated with apparent augmentation of SphK and S1PR expression in the lung [31]. In line with this, the expression of a mutated form of CFTR (dF508) promotes increased sphingolipid synthesis [32]. Interestingly, S1P controls its own degradation through CFTR conductance modulation, involving 5′ adenosine monophosphate-activated protein kinase (AMPK) and S1PR2 [33]. Together, these findings suggest CFTR functionality as a possible feedback system to modulate S1P metabolism. 

Previously, we validated CFTR’s contribution to pathological S1P signaling in the systemic and cerebral vasculature during experimental HF and reported a reduction of CFTR protein expression in several HF target organs, including the heart, the brain, and the lungs [25,27]. The link between HF-associated CFTR downregulation and S1P tissue levels, however, has not been elucidated yet. Here, we set out to investigate the relationship between S1P and dysfunctional CFTR in the HF lung.

## 2. Results

### 2.1. Impaired Pulmonary CFTR Expression during Heart Failure Links to Increased Sphingosine-1-Phosphate Concentrations in the Lung

**Figure 1:**
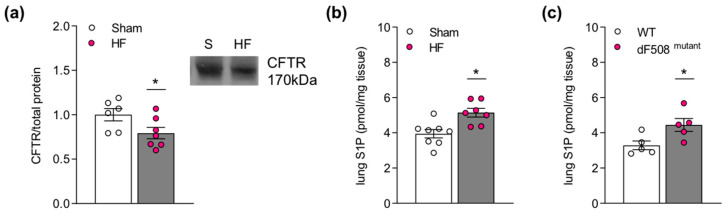
Impaired pulmonary cystic fibrosis transmembrane regulator expression during heart failure links to increased sphingosine-1-phosphate concentrations in the lung. (**a**) CFTR protein expression in lung tissue of HF mice and sham-operated controls determined with Western blotting. Inset showing representative CFTR protein expression pattern. (**b**) S1P levels in lung tissue of HF mice and sham-operated controls assessed by mass spectrometry. (**c**) Pulmonary S1P levels of CFTR mutant mice (dF508 mutation) and littermate controls assessed by mass spectrometry. Data are expressed as mean ± SEM, *t*-test where * denotes *p* ≤ 0.05. *CFTR*—*cystic fibrosis transmembrane regulator; dF508*—*delta F508 CFTR mutant; HF*—*heart failure; S*—*sham; S1P*—*sphingosine-1-phosphate; WT*—*wild-type*.

In a murine model of HF (i.e., 12-weeks post-myocardial infarction induced by permanent left anterior descending coronary artery ligation; average ejection fraction of 46.7 ± 7.5%), we observed significantly reduced pulmonary CFTR protein expression (Figure 1a), which was accompanied by an elevation of overall pulmonary S1P levels (Figure 1b). This HF-associated S1P elevation in the lung was not accompanied by altered S1P generating or degrading enzyme expression (Appendix A, Figure A1). The elevation of pulmonary S1P levels observed in dF508 mutant mice (Figure 1c), where defective CFTR folding impairs its biosynthetic and endocytic processing as well as its chloride channel function [34] and, hence, its S1P transporting capacity [25,33], suggests a link between impaired CFTR function and elevated S1P tissue levels in the lung.

### 2.2. Therapeutic CFTR Correction Attenuates Heart Failure-Associated Sphingosine-1-Phosphate Level Elevation in the Lung

**Figure 2:**
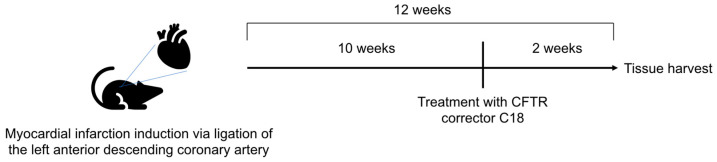
Experimental timeline. Male C57Bl/6N WT mice were subjected to myocardial infarction by permanent left anterior descending coronary artery ligation. Ten weeks post-myocardial infarction, mice received daily intraperitoneal injections of C18 (3 mg/kg BW) or an equivalent volume of vehicle for two consecutive weeks. Tissue was harvested and subjected to different experimental procedures. *BW*—*body weight; CFTR*—*cystic fibrosis transmembrane regulator; WT*—*wild-type*.

We next tested whether therapeutic correction of CFTR expression in vivo affects pulmonary S1P levels by treating HF mice with the CFTR corrector compound C18, starting 10 weeks post-myocardial infarction (Figure 2). Previous validation showed direct interactions of C18 with wild-type CFTR that leads to its stabilization and thereby increases CFTR expression at the plasma membrane [35].

**Figure 3:**
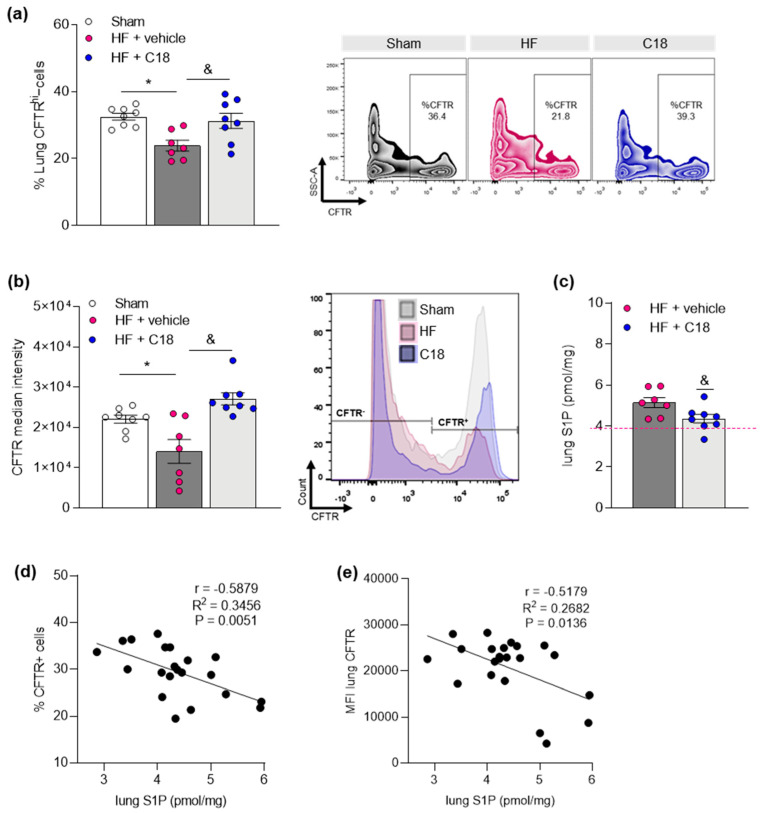
Correcting cystic fibrosis transmembrane regulator expression attenuates heart failure-associated pulmonary sphingosine-1-phosphate elevation. (**a**) Proportion of CFTR^+^ cells in the lungs of sham and HF mice after two weeks of vehicle or C18 treatment determined by flow cytometry. Representative zebra plots showing proportion of CFTR^+^ cells in lung tissue. (**b**) MFI quantification of CFTR^+^ cells in lung tissue of sham and HF mice after two weeks of vehicle or C18 treatment determined by flow cytometry and representative histograms. (**c**) Pulmonary S1P tissue concentrations of vehicle and C18-treated HF mice. The pink dotted line indicates sham levels. (**d**) Linear regression showing associations between the proportion of CFTR^+^ cells in lung tissue and pulmonary S1P levels. (**e**) Linear regression showing associations between the MFI of CFTR^+^ cells in lung tissue and pulmonary S1P levels. Data are expressed as mean ± SEM. In (**a**,**b**), one-way ANOVA with Tukey’s post hoc testing where * denotes *p* ≤ 0.05 between sham and HF + vehicle and & denotes *p* ≤ 0.05 between HF + vehicle and HF + C18; in (**c**) *t*-test where * denotes *p* ≤ 0.05; in (**d**,**e**), linear regression and Pearson’s correlation with exact r- and *p*-value computation. *CFTR*—*cystic fibrosis transmembrane regulator; HF*—*heart failure; MFI*—*median fluorescence intensity; S1P*—*sphingosine-1-phosphate*.

Thereafter, we tested if therapeutic CFTR correction in vivo affected pulmonary CFTR expression. Using a flow cytometry approach [27], we observed an attenuation of the HF-associated reduction of CFTR^+^ cell proportions in the HF lung after C18 treatment (Figure 3a). Moreover, the analysis of median fluorescence intensity (MFI) revealed markedly higher levels in CFTR^+^ cells in the lungs of C18-treated compared to vehicle-treated HF mice (Figure 3b), suggestive of higher CFTR expression per CFTR^+^ cell following C18 treatment. Correspondingly, pulmonary S1P concentrations were significantly lower in HF mice treated with C18 compared to vehicle-treated animals (Figure 3c). Correlation analyses revealed a significantly negative association between pulmonary S1P concentrations and the proportion of CFTR^+^ cells (Figure 3d) as well as the MFI of CFTR^+^ cells (Figure 3e) in the lung.

### 2.3. Therapeutic CFTR Correction Normalizes Heart Failure-Associated Elevation of Plasma Sphingosine-1-Phosphate Levels with Implications for Systemic Inflammation

**Figure 4:**
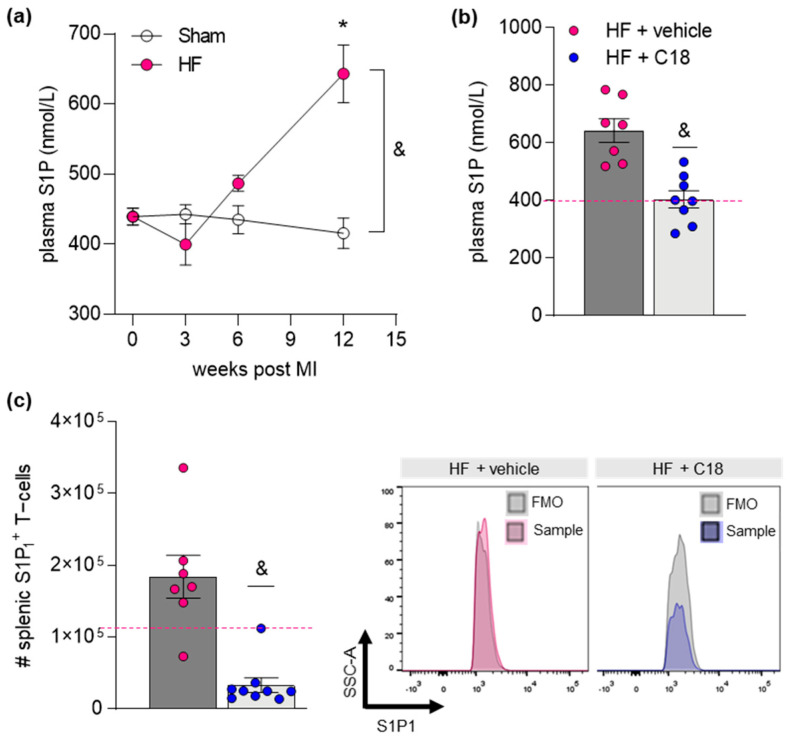
Correction of cystic fibrosis transmembrane regulator expression normalizes sphingosine-1-phosphate plasma levels with implications for systemic inflammation. (**a**) Plasma S1P concentrations during HF disease progression. (**b**) Comparison of S1P plasma levels between vehicle- and C18-treated HF mice. The pink dotted line indicates sham levels. (**c**) Frequency of splenic S1P_1_^+^ CD3^+^ T-cells assessed by flow cytometry in vehicle- and C18-treated HF mice. The pink dotted line indicates sham levels. Representative histograms showing the proportion of S1P1^+^ CD3^+^ T-cells in spleen tissue of HF mice after two weeks of vehicle (pink) or C18 (blue) treatment compared to their respective FMO controls (gray). In (**a**) two-way ANOVA where * denotes *p* ≤ 0.05 between baseline and different timepoints after Sidak post hoc testing; & denotes *p* ≤ 0.05 between sham and HF after Tukey’s post hoc testing; In (**b**,**c**), *t*-test where & denotes *p* ≤ 0.05. *CD*—*cluster of differentiation; FMO*—*fluorescence minus one, HF*—*heart failure; MI*—*myocardial infarction, S1P*—*sphingosine-1-phosphate; S1P_1_*—*S1P receptor type 1*.

In addition to lung tissue S1P, plasma S1P levels increased after surgery and were significantly elevated 12 weeks post-myocardial infarction when comparing to pre-surgery and sham levels in a longitudinal approach (Figure 4a). In the C18 group, treatment with CFTR corrector resulted in lower plasma S1P levels compared to the vehicle-treated HF group (Figure 4b). Considering the apparent chemotactic potential of S1P [5,9,36,37], we investigated the immune cell environment in secondary lymphoid tissue (i.e., spleen). In accordance with elevated plasma S1P, vehicle-treated HF mice presented with a significantly elevated number of splenic S1P_1_^+^ CD3^+^ T-cells (Figure 4c) among others (Table 1). HF mice that had received C18 treatment and presented with similar-to-sham S1P plasma levels revealed a significantly lower number of splenic S1P_1_^+^ CD3^+^ T-cells (Figure 4c) and other S1P_1_^+^ immune cells (Table 1).

### 2.4. Therapeutic CFTR Correction Attenuates Heart Failure-Associated Pulmonary Inflammation

**Figure 5:**
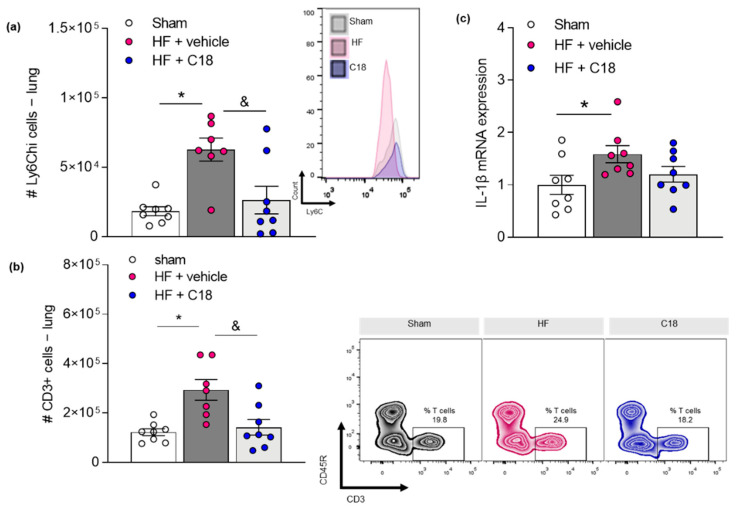
Correcting cystic fibrosis transmembrane regulator expression attenuates heart failure-associated inflammation in the lung. (**a**) Frequency of pro-inflammatory Ly6C^hi^ monocytes and (**b**) CD3^+^ T-cells in lung tissue of sham controls, and vehicle- and C18-treated HF mice determined by flow cytometry. Representative histogram or zebra plots showing the proportion of Ly6C^hi^ monocytes or CD3^+^ T-cells per 10^6^ viable CD45^+^ cells in digested lung tissue of sham mice and HF mice after two weeks of vehicle or C18 treatment. (**c**) mRNA expression of IL-1β in lung tissue of vehicle- and C18-treated HF mice compared to sham-operated controls. Data are expressed as mean ± SEM, one-way ANOVA with Tukey’s post hoc testing where * denotes *p* ≤ 0.05 between sham and HF + vehicle and & denotes *p* ≤ 0.05 between HF + vehicle and HF + C18. *CD*—*cluster of differentiation; HF*—*heart failure; IL-1β*—*interleukin 1 beta; Ly6C*—*lymphocyte antigen 6 complex, locus C; Ly6G*—*lymphocyte antigen 6 complex locus G*.

Following, we investigated if therapeutic CFTR correction and, thus, normalization of systemic and pulmonary S1P concentrations affected HF-associated inflammation in the lung [27]. C18 treatment attenuated the HF-associated accumulation of pro-inflammatory Ly6C^hi^ monocytes (Figure 5a) as well as CD3^+^ T-cells (Figure 5b) in lung tissue. Monocytes are major producers of interleukin 1 beta (IL-1β), which has powerful pro-inflammatory capacities [38]. It is a key player in airway inflammation [39] and lung fibrosis [40] and is also associated with worse prognosis in HF patients [41]. Statistical analysis of Il1b gene expression in lung tissue disclosed significant differences between sham-operated controls and vehicle-treated HF mice but not between sham-operated controls and C18-treated HF mice. However, no statistical difference was obtained between the two HF groups (Figure 5c; *p* = 0.4517). 

## 3. Discussion

Here, we show an inverse relationship between pulmonary S1P levels and CFTR expression in the HF lung with consequences for tissue-specific inflammation. HF progression is accompanied by steady increases of systemic S1P plasma levels and an augmentation of immune cells expressing S1P_1_, which critically controls S1P-mediated chemotaxis and, hence, might promote tissue inflammation during HF. CFTR corrector therapy attenuates HF-associated systemic and lung-specific S1P augmentation and results in lower S1P_1_ positivity of several splenic immune cell subsets, suggesting an important role for the CFTR-S1P axis in HF-mediated pulmonary inflammation. 

Previous studies have proposed a link between sphingolipid metabolism and CFTR. Specifically, CFTR-mediated S1P uptake was reported to be higher in cells expressing wild-type CFTR compared to cells expressing a mutated form of CFTR (dF508-CFTR) [26]. In its role as a critical bottleneck for S1P degradation, lower CFTR availability would therefore directly affect S1P levels. The herein presented results verify such an association during HF by showing an inverse relationship between CFTR expression and compartment-specific S1P levels (i.e., in the HF lung). In contrast to this, Halilbasic et al. reported lower plasma S1P levels in cystic fibrosis patients compared to healthy controls [42], however, the researchers found higher concentrations of unbound S1P in patients with the dF508-homozygous mutation compared to patients with a heterozygous dF508 CFTR mutation. It is important to note that the functional properties of ‘free’, unbound S1P are often different from those of S1P associated with its carriers, such as high-density lipoprotein, Apolipoprotein M or albumin (summarized in [43]). In the current study, we assessed total S1P concentrations and found significant differences in both plasma and lung tissue 12 weeks post-myocardial infarction. Nonetheless, it would be interesting to investigate whether S1P-carrier binding profiles are altered during HF disease progression as this may be important for human disease where multimorbidity may affect S1P binding and thereby its responses. 

A more regulatory function of CFTR within the S1P signaling axis was confirmed by studies that showed an apparent CFTR-S1P interplay in hypoxic pulmonary artery constriction mediated through transient receptor potential canonical 6-specific calcium mobilization [28] and an augmentation of S1P_2_-mediated vasoconstriction in brain and mesenteric resistance arteries [8,25]. For the latter, a similar relationship was identified in human mesenteric and skeletal muscle arteries [44]. With respect to CFTR functionality, S1P was shown to transiently inhibit CFTR activity via AMPK signaling [33]. Here, the rapid S1P-mediated inhibitory effect is transient and correlates with CFTR serine residue 737 (S737) phosphorylation. Considering these findings, it is tempting to speculate that elevated S1P levels fuel a vicious cycle of CFTR impairment-related S1P augmentation in the HF lung by continuously disturbing CFTR maturation and expression, plasma membrane localization or channel conductance alterations. In line with this, elevated pulmonary S1P levels were reported in patients with severe COPD [45], a disease with acquired CFTR dysfunction [29,46,47,48]. To date, only very few studies report S1P levels in cystic fibrosis (i.e., autosomal recessive disease with different CFTR phenotypes) with conflicting results [49,50]. Several chronic lung diseases, including asthma [51], pulmonary hypertension [52], and pulmonary fibrosis [53,54], however, have been linked to CFTR dysfunction [46,55,56] as well as elevated lung tissue S1P levels that accompany airway remodeling and inflammation. 

Mounting evidence suggests CFTR involvement in immune cell functions as CFTR deficiency, specifically in dendritic cells, augments the expression of several pro-inflammatory cytokines and impairs inflammation resolution [57]. Particularly in the lung, a dysfunctional myeloid cell milieu might seriously affect susceptibility to infection due to impaired clearance capacity. Given the importance of CFTR functionality for proper lung function, acquired alterations in CFTR expression and function resulting from different triggers, including pro-inflammatory cytokines [25,58,59], smoking [29,60,61], or S1P [33] may lead to a continuous cycle of lung tissue inflammation propagation. Lower pulmonary CFTR expression during HF that is accompanied by increased S1P levels in the HF lung and an elevation of pro-inflammatory monocytes and augmented IL-1β gene expression supports a critical interplay between CFTR and inflammation in the lung. Specifically, reduced expression of CFTR could lead to impaired S1P uptake and degradation; thus, more S1P would be available for S1P_1_-mediated immune cell chemotaxis to promote infiltration of circulating immune cells that add to an exacerbated cycle of inflammation. We recently observed that CFTR downregulation in the HF lung associates with an augmentation of TNF-α [27], a pro-inflammatory cytokine, which is known to induce M1-like macrophage phenotypes [62] and is secreted by classically polarized CD80^+^ macrophages [63] and monocytes [64]. The latter accumulate in the lung in our model [27]. Together, these data suggest that CFTR might play an important role in S1P tissue homeostasis necessary for maintaining an appropriate immune milieu in the HF lung. This is supported by findings showing decreased levels of S1P in broncho-alveolar lavage (BAL) fluid of CFTR knock-out animals that associate with dysfunctional dendritic cell function and, hence, increased susceptibility to infections [65]. S1P supplementation or abrogation of S1P degradation restored dendritic cell function and decreased infection-associated lung inflammation [49,65]. How, in particular, BAL fluid-specific S1P levels are maintained, and whether different S1P compartments in the lung interact, is currently unclear. Our attempts to determine S1P in BAL fluid in our model revealed extremely low S1P levels that were only detectable after prior concentration. We did not find statistically significant differences in BAL fluid S1P levels in HF mice compared to sham controls (data not shown). 

It is well-established that S1P plays a major role in the promotion of inflammation by modulating lymphocyte trafficking, calcium homeostasis, cellular growth, death, and differentiation, and activation of immune cells. Specifically, the concept of S1P-mediated immune cell chemotaxis has been exploited therapeutically in the clinic where the S1P_1_ antagonist FTY720 attenuates systemic and tissue inflammation during multiple sclerosis by preventing immune cell mobilization from lymphoid tissues [1]. CFTR corrector-mediated normalization of circulating S1P levels and a concomitant reduction of the frequency of S1P_1_^+^ immune cells in the spleen together with a lowering of pro-inflammatory monocytes and T-cells infiltrating the lung during HF support a role for S1P chemotaxis in HF-associated systemic and tissue inflammation. The regulation of S1P homeostasis at the systemic level is rather complex as several different cell types supply the pool of circulating S1P and control tissue S1P concentrations. In the blood, erythrocytes and platelets serve as the main S1P sources [66,67,68]. Particularly, the observed lowering of S1P plasma levels after CFTR corrector therapy is intriguing as most of the main suppliers of the circulating S1P pool express CFTR [69] but do not require CFTR function for S1P degradation [69,70]. Of interest, however, is a study showing that platelets respond with exaggerated activation when depleted of CFTR [70], which is rather suggestive of CFTR effects independent of S1P degradation. This is particularly important for HF where platelet abnormalities are well-described. The link between platelet activation and S1P levels, however, necessitates detailed mechanistic investigation. In addition to hematopoietic cells, endothelial cells provide approximately 40% of the total S1P plasma concentration through different mechanisms [71,72]. Like erythrocytes and platelets, endothelial cells also express CFTR. But in contrast to the other two S1P pool supplying cell types, endothelial cells, including pulmonary endothelial cells, possess a high capacity to degrade S1P [73], which may be altered during disease. In response to shear stress, for instance, endothelial cells decrease their expression of S1P degrading enzymes [74]. It is therefore likely that endothelial CFTR may be needed for effective intracellular S1P degradation and, thus, may be targetable with CFTR corrector therapy. 

## 4. Conclusions

**Figure 6:**
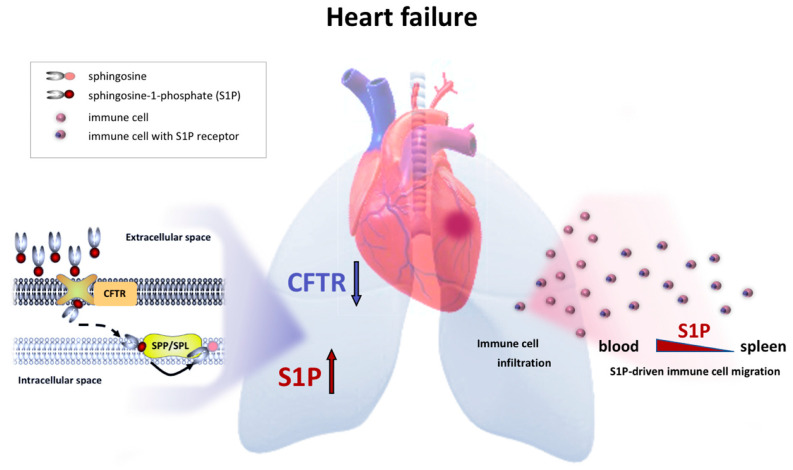
Schematic overview of heart failure-associated sphingosine-1-phosphate responses in the lung and the blood. HF induced by myocardial infarction is accompanied by increased pulmonary S1P levels resulting from a reduction of cell surface expression of CFTR in the lung. In its role as cellular S1P import mechanism, impaired CFTR expression limits intracellular S1P degradation by S1P phosphatase or S1P lyase [25], leading to an accumulation of tissue S1P. Additionally, HF promotes S1P plasma level elevation and enhanced immune cell egress of S1P1+ cells from lymphoid tissue (e.g., spleen) into the bloodstream from where they can migrate (dependent and independent of S1P) into the lung and thereby contribute to hyperinflammation in the lung during HF. *CFTR*—*cystic fibrosis transmembrane regulator; HF – heart failure, S1P*—*sphingosine-1-phosphate; S1P1*—*S1P receptor 1; SPL*—*S1P lyase; SPP*—*S1P phosphatase*.

HF associates with an augmentation of systemic and lung-tissue-specific S1P levels that link to a pro-inflammatory environment in the lung (Figure 6). Alterations of S1P_1_ positivity of several immune cell subsets suggest S1P involvement in immune cell egress from lymphoid tissue during HF with consequences for tissue inflammation, specifically in the lung. Our data further suggest an intimate link between lung tissue expression of CFTR, which, in its role as an important S1P import mechanism, may critically control pulmonary S1P levels and thereby drive S1P-mediated immune cell infiltration into the lung. Supportive of this paradigm are our findings showing that therapeutic CFTR correction attenuates the HF-associated elevation of systemic and pulmonary S1P concentrations, reduces the frequency of S1P_1_ positivity on immune cells and, hence, lowers the percentage of lung infiltrating immune cells. Together, these results are suggestive of CFTR as a therapeutic target in HF-associated lung inflammation.

## 5. Future Perspectives

A genetic CFTR defect has been studied in the context of cystic fibrosis for many decades, but recently it was shown that other chronic lung diseases, including COPD and asthma, present with acquired CFTR dysfunction [46,47,55]. Our previous and current studies extend these findings to target organ damage during HF. Our data provide important insights into acquired CFTR dysfunction that may be relevant for different diseases. CFTR is widely expressed throughout the body (e.g., brain, lung, intestine, etc.) [25,75] and has been reported to be affected during various disease states [11,29,55,61,76], which may make CFTR a yet underexplored target for treatment in diseases other than cystic fibrosis. Similar to trials that verified Ivacaftor efficacy in COPD [77], our studies validate benefits of the CFTR modulator C18 in lung inflammation and cerebrovascular dysfunction [11] that associate with HF in our model. C18 has not yet been widely tested and more research on involved mechanisms and validation in human disease are warranted before clinical trials could be initiated. 

Specifically, with respect to the suggested link between CFTR and S1P signaling, further work needs to determine: (1) the source of augmented S1P production during HF, and (2) if and how pulmonary and circulating S1P pools interact. In addition, studies need to investigate whether (pulmonary) endothelial cells supply the circulating S1P pool during HF and also facilitate the infiltration of peripheral immune cells into the lung as pulmonary endothelial cell-derived S1P affects endothelial cell migration [73] and, thus, may impair barrier function [78]. In light of this, defective CFTR function in endothelial cells has been shown to associate with endothelial activation and a persisting pro-inflammatory state of the endothelium with increased leukocyte adhesion [79]. Moreover, we have previously shown that S1P itself is capable of downregulating CFTR surface expression [33]. Considering endothelial cells as an important interface between circulating blood and tissue, it is likely that they play a critical role in our model by excessively supplying the circulating S1P pool to increase S1P-mediated immune cell egress from lymphoid tissue and by being a target of (S1P-dependent or independent) CFTR downregulation that affects barrier integrity and, thus, allows peripheral immune cells to enter target tissue, such as the lung during HF. These possible links between CFTR and S1P signaling are intriguing but require detailed cell type-specific investigation to mechanistically characterize the circulating and pulmonary CFTR-S1P axis during HF.

## 6. Materials and Methods

### 6.1. Chemicals and Reagents

All chemical reagents and solutions were purchased from Fisher Scientific (Gothenburg, Sweden), Saveen & Werner (Limhamn, Sweden) or Sigma-Aldrich (Stockholm, Sweden), unless otherwise stated. A commercially available primary antibody against CFTR (CF3) was used for Western blotting (see Appendix A, Table A1). An HRP-labelled secondary antibody against goat anti-mouse (Nordic Biosite, Sweden) was used for visualization. Primers for qPCR were purchased from Eurofins (Ebersberg, Germany). The present investigation acquired the CFTR corrector therapeutic “C18” through the *Cystic Fibrosis Foundation Therapeutics* Chemical and Antibody Distribution Programs (http://www.cff.org/research (accessed on 15 September 2018)). Dr. Robert Bridges (Rosalind Franklin University of Medicine and Science, Chicago, IL, USA) provided the C18 compound.

### 6.2. Animals 

This investigation conforms to the Guide for Care and Use of Laboratory Animals published by the European Union (Directive 2010/63/EU) and with the ARRIVE guidelines. All animal care and experimental protocols were approved by the institutional animal ethics committee at Lund University (Dnr.: 5.8.18-08003/2017, 5.8.18-04938/2021) and were conducted in accordance with European animal protection laws. Commercially available male wild-type mice (12–14 weeks; C57BL/6N) were purchased from Taconic (Lyngby, Denmark). All mice were housed under a standard 12 h/12 h light−dark cycle, fed normal chow, and had access to food and water ad libitum. HF animals were randomly assigned to vehicle or treatment groups. In order to obey the rules for animal welfare, we designed experimental groups in a way that minimizes stress for the animals and guarantees maximal information using the lowest group size possible when calculated with a type I error rate of α = 0.05 (5%) and power of 1 − β > 0.8 (80%) based on previous studies [8,9].

### 6.3. Induction of Myocardial Infarction in Mice 

Myocardial infarction was induced by surgical ligation of the left anterior descending coronary artery [8,9]. Briefly, mice were anaesthetized with isoflurane, intubated with a 22-gauge angiocatheter and ventilated with isoflurane (1.5–2% in room air) to maintain narcosis. Under sterile conditions, the thorax and pericardium were opened, and the left anterior descending coronary artery was permanently ligated with 7–0 silk suture (AgnThos; Stockholm, Sweden). In sham-operated controls, the thorax and pericardium were opened, but the left anterior descending coronary artery was not ligated. Following the procedure, the chest was closed, the mice were extubated upon spontaneous respiration and received 2 µL/g mouse Buprenorphine (0.05 mg/mL; Indivior, Dublin, Ireland) for 2–3 days according to an approved analgesia protocol. All experimental measurements in the HF model were conducted after 12 weeks post-myocardial infarction. 

### 6.4. Assessment of Cardiac Function Using Magnetic Resonance Imaging 

Cardiac function was assessed using magnetic resonance imaging on a 9.4 T MR horizontal MR scanner equipped with Bruker BioSpec AVIII electronics, a quadrature volume resonator coil (112/087) for transmission and a 20 mm linear surface loop coil for reception (Bruker, Ettlingen, Germany), operating with ParaVision 6.0.1. Mice were anaesthetized with isoflurane in room air with 10% oxygen and kept at a respiration of 70–100 bpm and at 36–37 °C body temperature. Flow compensated FLASH with ECG and respiration triggering (Stony Brook, NY, USA) with a resolution of 0.13 × 0.13 × 1 mm^3^ was used for all MR scans. Positioning of the cardiac images was achieved by three orientational scans: (1) three axial slices (TR = 50 ms, TE = 2.5 ms), (2) and (3) each with one slice (TR = 6 ms, TE = 2.1 ms, 24 timeframes) orthogonal to each other with slices positioned through the left and right ventricle and through the outflow tract of the left ventricle and the apex, respectively. Short axis view images of 9–10 slices (depending on heart size) were acquired with 24 timeframes in each (TR = 6 ms, TE = 2.1 ms). The short axis images were used for determination of the ejection fraction using the freely available software Segment version 3.0 R7820 (http://segment.heiberg.se (accessed on 16 May 2019) [80].

### 6.5. Fluorescence Activated Cell Sorting

Before euthanasia through decapitation, mice were sedated using inhalation anesthesia (isoflurane 2.5% at 1.5 L/min in room air). Whole blood was collected and the trachea cannulated. Spleen was extracted before trans-cardiac perfusion, whereafter the lung-heart block was extracted, and a broncho-alveolar lavage was performed by instilling 1 mL of sterile PBS. For flow cytometry experiments, the left lung was cut into pieces and enzymatically digested in a DNAse-collagenase XI mix under continuous agitation at 37 °C before the homogenate was passed through a 40 µm cell strainer. After centrifugation, red blood cells were lysed, and the cell pellets were incubated in Fc block prior to staining with antibodies (see Appendix A, Table A1). As for the spleen, red blood cells were lysed after homogenization of the tissue through a 40 µm cell strainer. Cell pellets were incubated in Fc block prior to staining with antibodies (see Appendix A, Table A1). Data acquisition was carried out in a BD LSR Fortessa cytometer using FacsDiva software Vision 8.0 (BD Biosciences). Data analysis was performed with FlowJo software (version 10, TreeStar Inc., Ashland, OR, USA). Cells were plotted on forward versus side scatter and single cells were gated on FSC-A versus FSC-H linearity. 

### 6.6. Western Blotting 

Lung samples (a part of the right middle lobe) were homogenized in 1× PBS using an Ultra-Turrax TP18-10 (Janke & Kunkel KG) and proteins lysed in RIPA buffer (25 mM Tris-HCl pH 7.6, 150 mM NaCl, 5 mM EDTA, 1% Triton X-100, 1% sodium deoxycholate, 0.1% SDS) supplemented with phosphatase and protease inhibitors. Samples were frozen at −80 °C and thawed on ice. Thereafter, protein extracts were cleared from insoluble material by centrifugation for 15 min at 20,000× *g* at 4 °C and stored at −20 °C thereafter. Protein content was measured using the Pierce™ BCA Protein Assay Kit according to the manufacturer’s instructions. A quantity of 10–30 µg protein was separated in SDS-PAGE prior to transfer onto PVDF membranes (VWR) using wet transfer. Membranes were blocked with 5% non-fat dry milk powder in PBS-T (1× PBS, 0.05% Tween 20) for 1 h at room temperature and incubated with primary CFTR antibody (CF3; see Appendix A, Table A1) overnight at 4 °C. Blots were incubated with secondary, HRP-labelled antibody (goat anti-mouse; see Appendix A, Table A1) for 2 h at room temperature. Enhanced chemiluminescence was used to visualize proteins and the signals were measured using a ChemiDocTM MP (Bio-Rad, Hercules, CA, USA). Protein expression was quantified in relation to total protein acquired with a stain-free approach and normalized to sham animals. 

### 6.7. Quantitative Real-Time PCR (qPCR) 

For total RNA isolation, a part of the right middle lobe was homogenized in 1 mL Trizol (Invitrogen, Waltham, MA, USA) using an Ultra-Turrax TP18-10 and isolated according to the manufacturer’s manual. 1 µg of mRNA was reverse transcribed into cDNA using the High-Capacity cDNA Reverse Transcription Kit in an T100TM Thermal Cycler (Bio-Rad, Hercules, CA, USA). The resulting cDNA was diluted 12.5× to a final volume of 250 μL, which was subsequently used as a template for PCR reactions. The PCR protocol consisted of 40 cycles of 30 s denaturation (95 °C); 45 s primer annealing (60 °C) and 45 s primer extension (72 °C) using a CFX384TM Real-Time System with a C1000 TouchTM Thermal Cycler (Bio-Rad, Hercules, CA, USA). A list of the primers utilized is provided in Appendix A, Table A2. All data were normalized to the species-specific housekeeping gene L14 and quantification was carried out via the absolute method using standard curves generated from pooled cDNA representative of each sample to be analyzed.

### 6.8. S1P Mass Spectrometry

S1P in plasma was quantified by liquid chromatography-coupled tandem mass spectrometry as described in [20]. From lung tissue homogenates (right inferior lobe), S1P was extracted using organic solvents as described in [81] after spiking homogenates with S1P-D7 (Avanti Polar Lipids/Merck, Darmstadt, Germany) as internal standard. Lipid extracts were dried in a nitrogen stream, taken up in methanol, and subjected to quantitative mass spectrometry analysis as above, applying a 6-point standard curve of extracts of 0.25–6 pmol S1P in fatty acid free BSA/PBS.

### 6.9. Statistics

All data are expressed as mean ± SEM, where N is the number of animals. Data were statistically analyzed using GraphPad Prism 8 software (San Diego, CA, USA). For longitudinal comparisons, two-way ANOVA with Sidak post hoc testing was used to assess the effects of experimental group and time post-surgery. For comparison of multiple independent groups, parametric one-way analysis of variance (ANOVA) was used, followed by Tukey’s post hoc testing, with exact *p* value computation. In case of non-normally distributed data (tested with Shapiro-Wilk test), the non-parametric Kruskal–Wallis test with Dunn’s post hoc testing and exact *p* value computation was used for multiple comparisons. For comparison of two groups, a two-tailed unpaired *t*-test was utilized. Differences were considered significant at error probabilities of *p* ≤ 0.05.

## Figures and Tables

**Table 1:** Number of splenic S1P_1_^+^ immune cell populations in vehicle- and C18-treated HF mice compared to sham-operated controls. Data are expressed as mean ± SEM. One-way ANOVA with Tukey’s post hoc testing where * denotes *p* ≤ 0.05 between sham and HF + vehicle/HF + C18 and & denotes *p* ≤ 0.05 between HF + vehicle and HF + C18. *CD3*—*cluster of differentiation 3; HF*—*heart failure; Ly6C*—*lymphocyte antigen 6 complex, locus C; S1P_1_*—*S1P receptor subtype 1*.

# S1P_1_^+^ Splenic Immune Cells	Sham	HF + Vehicle	HF + C18
CD3^+^ T-cells	10.7 × 10^4^ ± 0.61 × 10^4^	17.3 ×10^4^ ± 2.78 × 10^4^ *	2.1 × 10^4^ ± 0.23 × 10^4^ &
Ly6C^hi^ monocytes	1804 ± 291	3388 ± 1008	434 ± 39 &
Neutrophils	3288 ± 562	15,675 ± 4262 *	3026 ± 697 &

**Figure A1:**
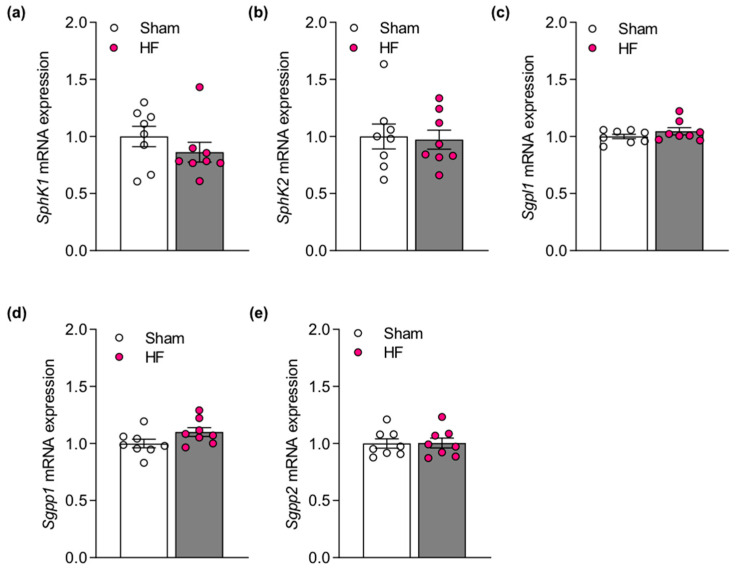
Gene expression of sphingosine-1-phosphate generating enzymes (**a**) SphK1 and (**b**) SphK2, and S1P degrading enzymes (**c**) Sgpl, (**d**) Sgpp1 and (**e**) Sgpp2 in the lungs of sham and HF mice. N = 8. *HF*—*heart failure; S1P*—*sphingosine-1-phosphate; SphK*—*sphingosine kinase, Sgpl*—*S1P lyase, Sgpp*—*S1P phosphatase*.

## Data Availability

The data presented in this study is available in the article and Appendix A.

## Appendix A

**Table A1:** Primary and secondary antibodies used for FACS and Western Blotting (WB).

Antibody	Company	Ordernr	Concentration and Application	Size [kDa]
Live/Dead Aqua	Invitrogen	L34966	1:500 FACS	
B220 AF488	R&D	FAB1217G	1:200 FACS	-
CD11b PE Tex Red	Invitrogen	RM2817	1:200 FACS	-
CD3 eFluor450	eBioscience	48-0032-82	1:200 FACS	-
CD45 AF700	R&D	FAB114N	1:200 FACS	-
CFTR	Thermo Fisher	MA1-935	1:400 FACS; 1:1000 WB	170
HRP-labeled goat anti-mouse	Cell signalling	7076S	1:10,000 WB	-
Ly6C PE-Cy7	eBioscience	25-5932-82	1:200 FACS	-
Ly6G APC	eBioscience	17-9668-82	1:200 FACS	-
S1P_1_ PE	R&D	FAB7089P	1:200 FACS	-

**Table A2:** Primers used for qRT-PCR. *L14—ribosomal protein L14; SphK1—sphingosine kinase 1; SphK2—sphingosine kinase 2; Sgpl—sphingosine-1-phosphate lyase; Sgpp1—sphingosine-1-phosphate phosphatase 1; Sgpp2—sphingosine-1-phosphate phosphatase 2; Il1b—interleukin 1 beta*.

Gene	Sequence (5′ to 3′)	Annealing Temp. [°C]
*L14*	Fw: GGCTTTAGTGGATGGACCCT Rv: ATTGATATCCGCCTTCTCCC	59.4 57.3
*SphK1*	Fw: GGTGAATGGGCTAATGGAACG Rv: CTGCTCGTACCCAGCATAGTG	59.8 61.8
*SphK2*	Fw: CACGGCGAGTTTGGTTCCTA Rv: CTTCTGGCTTTGGGCGTAGT	59.4 59.4
*Sgpl1*	Fw: GGTGTATGAGCTTATCTTCCAGC Rv: CTGTTGTTCGATCTTACGTCCA	60.6 58.4
*Sgpp1*	Fw: GAGCAACTTGCCGCTCTACTA Rv: GGTCGAGATTCCAGATCCAGAA	59.8 60.3
*Sgpp2*	Fw: GTTCTCTACGCTGGTGTGTCT Rv: GCAGGGTAGGTCAGAGCAAT	59.8 59.4
*Il1b*	Fw: GAAGAGCCCATCCTCTGTGA Rv: TTCATCTCGGAGCCTGTAGTG	59.4 59.8
